# Bernard and Robin on the Blood

**Published:** 1854-12

**Authors:** 


### BOOK NOTICES.

*Bernard & Robin on the Blood*, *by* Atlee. Philadelphia: Lippincott, Grambo & Co., 1854: from the Publishers.

Dr. Atlee has published his notes of M. Bernard's lectures during the winter of 1853—54, with an appendix, containing some notes of the lectures of M. Charles Robin, on the anatomy of the blood, of the blood vessels, and of the parenchymatous organs.

In looking over this little book, we find but little that is really new to American readers. The numerous letter writers at Paris during the last few years have kept the profession well informed in reference to the improvements in French science, and there seems to be but little wanting, except a systematic arrangement and classification of the facts which have been floating in our periodical literature. This, so far as the blood is concerned, we were prepared to expect in Dr. Atlee's book. Such however is not the case, perhaps necessarily from the fact that the notes were hastily taken at the time of the lectures. There is however an abundance of interesting facts, many of them of much practical value. A few of these we shall notice. Our readers are aware that one theory of the circulation is based on the hypothesis, that the blood receives an increase of temperature while passing through the lungs. This, we believe, is the fundamental idea of the *filia nata jovis* of Dr. Cartwright, although the Dr. himself repudiates the doctrine on this subject. We quote the following:

"While at the extremities the temperature varies, for certainly it is lower in the fingers in winter than in summer, in the heart it is fixed. In the right ventricle it is higher than in the left. The contrary was long supposed to be the fact, from the experiments being made on the animal after death, when the heat was leaving the body, and the right ventricle being thinner than the left, of course it cools faster. The heart being withdrawn from the body, put a thermometer in each ventricle, and place the organ in water at 40° Centig, the thermometer will stand at 40° also; then remove it and leave it in a temperature of 16°, and in a short time there will be a difference of 6° in the two instruments. The difference of temperature then noted by these experiments was not owing to a difference previously existing in the blood. M. Bernard last summer experimented upon at least a dozen animals while yet alive, the instruments being passed into the brachio-cephalic artery and into the descending vena cava. The same thermometer was used, for fear of the possibility of a slight difference in instruments, and the experiment was varied in every possible way. The temperature was always higher in the right ventricle, where it was 39° or 40°. The difference (and the thermometer used was graduated to the hundredth of a degree) was never more than one-half or one third of a degree. As will be seen hereafter, the maximum of temperature is in the portal vein. This diminution of temperature is owing to the air, which cools the blood; when it does so in the fingers, why should it not do so in the lungs. M. Bernard has made his experiments in the summer when the weather was very warm, and will repeat them in order to see if there be a diminution, proportionate to the temperature of the air. To resume; the blood of mammiferous animals is warmer in the veins than in the arteries; in the portal vein, than in the aorta."

In reference to the coagulation of the blood we find the following:—

"In some circumstances the blood coagulates with great difficulty, as after certain poisons, and in animals exhausted by fatigue; and cold produces the same effect. If an animal be made to die slowly by cold, and just before death some blood be taken, it will scarcely coagulate. M. Bernard does not know what becomes of the fibrine in these cases, but notices the fact. When blood is taken from cold-blooded animals in the winter, it coagulates with great difficulty, and sometimes not at all; when taken in the summer it coagulates perfectly.

M. Bernard thinks that this coagulability is connected with the respiration, with the animal temperature; that it is proportionate to the elevation of temperature, as is shown by observations on birds, mammiferous animals, &c., and also, that in the individual, it is influenced by cold."

Is seems to us that we may account for the phenomena here observed on the hypothesis that fibrine is produced in the process of secondary assimilation and that it sustains to certain tissues the relation of excretable material, while to other structures it may subserve the purpose of nutrition. The manner in which excretions retained in the system through functional or organic lesion of their proper organs are finally removed, is beautifully shown in the following experiment :

A singular fact noticed after the removal of the kidneys is this. In twenty-four hours about six grammes of urea and uric acid are produced in an animal, and yet if you bleed at the end of twenty-four hours after the operation, you will still find them to be in very feeble proportion in the blood. Where is it eliminated in these early times of the operation? It is in the intestines, and particularly in the gastric juice, as a fistula in the stomach of a dog enabled M. Bernard to demonstrate. Before the operation there was none in what issued from the opening, and immediately after there was. After remaining for some time in the intestine, the urea changed into ammonical salts, and what is strange, the gastric juice continued all the time to be secreted. When substances generally eliminated by the kidneys were injected into the blood, they were found in the stomach. In lower animals where there is no kidney, it is in this manner that the elimination always takes place. This operation does not much enfeeble the animal. He vomits, and has diarrhœa, and at last, when enfeebled from not eating, he becomes sick. At this time the urea is no longer eliminated by the intestine, and you find much in the blood. And thus, in individuals affected with disease of the kidneys, you often see vomitings and derangement of digestion, owing to the urea being thrown into the intestines, and there undergoing change into ammoniacal products. In diabetes you find very little urea in the urine, and M. Sigalas gave urea to such patients in the expectation of finding it there, but he did not find the quantity to be increased ; it was transformed into ammoniacal products.

M. Bernard goes on to examine the blood of the portal system, and the circumstances modifying it, the notes however contain but little if anything that is new. His discovery of the function of the liver as a manufactory of sugar is dwelt upon at some length.

M. Ro in has until quite recently denied that the red corpuscles of human blood ever contain nuclei ; he has however recently discovered them in the blood of a very young human embryo. In the following letter to the author, Dr. Atlee, he makes the announcement.

"Since your departure, I have had the opportunity of obtaining four human embryos, perfectly fresh ; their lengths were 3 millimetres, 16 millimetres, and 25 millimetres. I made some interesting observations upon them, relative to all their tissues, and particuliarly to their blood. I send you, here, the *resume*, which you can translate and publish, if you think proper.

"The majority of the red globules of the blood of embryos are from 8 to 12 milliemes of a millimetre broad, and some of them are even 13 and 14 milliemes. The most of them also have one, or rarely, two nuclei. These nuclei are spherical, finely granular, from 3 to 4 milliemes of a millimetre broad; but the globules of the blood are *bi-concave* as the globules of the adult. In embryos, 25 millimetres long, already *not more than one half* of the globules have a nucleus, the others have no nucleus, and are of about the same size as the globules of the adult, that is to say 8 milliemes of a millimetre. The nucleus is rarely in the centre of the globule, but almost always a little to the side of the central concavity of each face, and they do not prevent the globule from having the flattened form of the globules of the adult. These globules fold upon themselves, or become dentated; with the greatest facility, as soon as the serum is a little changed. At the same time many among them take an ovoid form, but not at all an oval-flattened; a fact which easily distinguishes them from those of fish and of reptiles, which are oval and flattened. This form, which M. Vance told me he had seen with a professor of Vienna, does really exist, but it is an accidental deformity, taking place with facility from alterations in the serum, in the same way as the foldings and other irregularities of which I have spoken.

"The globules of the embryo, seen in a serum unchanged or but slightly modified, are elastic, and resume with rapidity their form, when, having been deformed by compression, they cease to be compressed. They often assume a very elegant form, when they are accumulated and reciprocally compressed.

"The nuclei of the red globules are unaffected by acetic acid and water, but the mass of the globule is affected as that of the globule of the adult.

"I was able to verify with ease, on these embryos, that the red globules are in no way derived from the white globules, which act very differently, have another structure, &c.

"In embryos from 40 to 50 centimetres (millimetres ?) long, the globules have already the volume, which they will always have, and no longer have nuclei. The ovoid form of which I have spoken above is generally less regular than those I have represented."

In a note on tubercle a brief but clear description is given of the microsposic character of the tubercular corpuscle.

"These corpuscles are bodies slightly irregular on the surface, and containing no nucleus. They are polyhedric, either of equal diameters, or elongated; when elongated, they are not flattened. Their borders are dentated, and in the centre are many granulations, contained in an amorphous mass. Their diameter is from 0.007 to 0.009 millimetres, very rarely 0.010; that is to say, though such almost always exist in a specimen, they are few in number. They are very rarely rounded; when nearly so you find them slightly dentated. Their contour is well marked, quite dark. Water has no action upon them; acetic acid pales but does not dissolve them: this it is important to note, for it is thus easy to distinguish them from nuclei of any kind, for all nuclei except those of cancer are rendered darker by it. The corpuscle contains no nucleus itself, and thus can easily be distinguished from concrete pus-globules, in which acetic shows at once the nuclei.

"In addition to this, the corpuscle of tubercle is much less paled.

"Their structure is simple, an amorphous matter sprinkled with granulations. In some are found fatty granulations, that could be taken for nuclei."

In conclusion we must say that we are disappointed in the work. Perhaps we should be equally disappointed should we listen to the lectures of M. Bernard himself. It is to what an author writes, not to what he says that we must look for a correct exponent of his views.

Men are comparatively careless both of method and fact, when there is no record of their language, for this reason if for no other, we would be glad to see our public teachers write their lectures. The might possibly be less imaginative and adorned with fewer rhetorical beauties, but we think they would be more accurate and more systematic in their arrangement; two qualities of essential importance to the student.

